# Comparison between Prostaglandin E-major urinary metabolite and C-reactive protein levels to reflect endoscopic scores in patients with ulcerative colitis

**DOI:** 10.1038/s41598-021-95761-6

**Published:** 2021-08-10

**Authors:** Natsuki Ishida, Satoshi Tamura, Takahiro Miyazu, Shinya Tani, Mihoko Yamade, Moriya Iwaizumi, Yasushi Hamaya, Satoshi Osawa, Takahisa Furuta, Ken Sugimoto

**Affiliations:** 1 First Department of Medicine, Hamamatsu University School of Medicine, 1-20-1 Handayama, Hamamatsu-shi, Higashi-ku, Shizuoka, 431-3192 Japan; 2 Department of Laboratory Medicine, Hamamatsu University School of Medicine, 1-20-1 Handayama, Hamamatsu-shi, Higashi-ku, Shizuoka, 431-3192 Japan; 3 Department of Endoscopic and Photodynamic Medicine, Hamamatsu University School of Medicine, 1-20-1 Handayama, Hamamatsu-shi, Higashi-ku, Shizuoka, 431-3192 Japan; 4 Center for Clinical Research, Hamamatsu University School of Medicine, 1-20-1 Handayama, Hamamatsu-shi, Higashi-ku, Shizuoka, 431-3192 Japan

**Keywords:** Inflammatory bowel disease, Ulcerative colitis

## Abstract

Prostaglandin E-major urinary metabolite (PGE-MUM) and C-reactive protein (CRP) are useful biomarkers in patients with ulcerative colitis. However, whether changes in endoscopic scores over time are reflected in the values of these biomarkers has not been verified. This prospective observational study aimed to assess the relationship between changes in biomarker levels and endoscopic scores in patients with ulcerative colitis. A total of 100 colonoscopy intervals of patients with ulcerative colitis were enrolled. The relationship between variations in the Mayo endoscopic subscore over time and the accompanying changes in biomarker values were investigated. PGE-MUM levels showed a significant rise in the increased endoscopic score group (P = 0.007) and a decrease with reduced endoscopic score group (P = 0.023). CRP levels showed a significant decline with lower endoscopic values (P < 0.001); however, there was no corresponding increase with higher endoscopic scores (P = 0.141). Biomarker levels remained unchanged with stable endoscopic scores (P = 0.090 and P = 0.705). PGE-MUM levels varied significantly, and corresponded to the mucosal healing state (P = 0.019 and P = 0.009). The correlation between changes in PGE-MUM and the endoscopic score was stronger than that for CRP (r = 0.518, P < 0.001 vs. r = 0.444, P < 0.001, respectively). PGE-MUM reflected changes in endoscopic scores more accurately than CRP.

## Introduction

Ulcerative colitis (UC) is a chronic idiopathic inflammatory bowel disease (IBD) characterized by symptoms such as diarrhoea, abdominal pain, and haemorrhagic stools^1^. Initially, the primary goal of medical therapy for UC was to achieve stable clinical remission. However, with the development of various evaluation and treatments methods, the current objective is to achieve mucosal healing (MH), which has been proven to improve the prognosis of UC^2–6^. Although endoscopic evaluation is typically required to determine the state of MH, several biomarkers have been reported to be effective alternatives. C-reactive protein (CRP) is a common long-standing inflammatory biomarker. Although CRP is not disease-specific, it is widely used for monitoring UC in clinical practice because it is easy to measure^7–12^.

Faecal calprotectin (FC) has recently been reported as an extremely significant biomarker that reflects endoscopic scores^12–17^, and the faecal immunochemical test (FIT) has been described to be reflective of the mucosal state in patients with UC^18–23^. The usefulness of serum leucine-rich alpha-2 glycoprotein (LRG) has also been reported and LRG is used in clinical practice^24^. Using biomarkers to evaluate UC disease activity may reduce the number of colonoscopies (CS) performed, as well as minimize CS-related complications, such as bowel perforation. Additionally, fewer colonoscopies may also prevent infectious diseases, such as the coronavirus disease (COVID-19), from being transmitted during endoscopic examination^25^.

Prostaglandin E-major urinary metabolite (PGE-MUM) has been reported as a novel and effective biomarker for UC^26^. PGE-MUM is sourced from urinary specimens^27^ and has been reported to be useful in paediatric patients with UC^28^. Additionally, PGE-MUM may be efficient in distinguishing between chronic enteropathy associated with the solute carrier organic anion transporter family member 2A1 (SLCO2A1) gene and Crohn's disease^29^. Compared with FIT, PGE-MUM is particularly effective in patients with long-term UC^30^ and predicts possible relapse^31^.

Multiple cross-sectional studies have examined the correlation between biomarkers and endoscopic scores in patients with UC. The receiver operating characteristic curves for the prediction of achieving MH with certain biomarkers have been analysed. However, only a few longitudinal observational studies have investigated the changes in biomarkers in relation to the endoscopic scores of patients with UC. If the exacerbation or improvement of colon inflammation can be confirmed using biomarkers, an appropriate treatment plan can be selected accordingly. Hiraoka et al., demonstrated that a variation in precedent and subsequent endoscopic scores resulted in significant variations in FIT and FC^23^. However, studies investigating changes in PGE-MUM in relation to changes in endoscopic scores of UC patients have yet to be reported.

In this study, changes in endoscopic scores and accompanying variations in the PGE-MUM and CRP values of UC patients were longitudinally analysed.

## Results

### Patient characteristics

The characteristics of the patients with UC that were enrolled at precedent CS (N = 100) are shown in Table 1. The mean patient age was 49.7 years, and the mean disease duration was 8.3 years (range 0.3–37 years). The mean PGE-MUM and CRP levels were 26.9 µg/g·Cr and 0.38 mg/dL, respectively. The mean interval between precedent and subsequent colonoscopies was 14.9 months.

**Table 1:** Patient characteristics.

Characteristics at precedent CS	N = 100
Age (years), mean (range) ± s.d	49.7 (18–84) ± 15.6
Male/Female, n (%)	66/34 (66.0/34.0)
Disease duration (years), mean (range) ± s.d	8.3 (0.3–37) ± 8.8
**Disease extent, n (%)**	
Extensive colitis	59 (59.0)
Left-sided colitis	31 (31.0)
Proctitis	10 (10.0)
CAI (Rachmilewitz index), mean (range) ± s.d	1.7 (0–17) ± 2.5
PGE-MUM (µg/g·Cr), mean (range) ± s.d	26.9 (5.1–101.0) ± 17.3
CRP (mg/dL), mean (range) ± s.d	0.38 (0.01–6.59) ± 0.94
**MES, n (%)**	
MES 0	37 (37.0)
MES 1	28 (28.0)
MES 2	31 (31.0)
MES 3	4 (4.0)
Interval period between CS (months), mean (range) ± s.d	14.9 (0.1–48.0) ± 8.4
**Medication, n (%)**	
Oral 5-ASA	67 (67.0)
Suppository 5-ASA	11 (11.0)
Systemic steroids	17 (17.0)
Immunomodulators	37 (37.0)
Biologics	27 (27.0)

*5-ASA* 5-aminosalicylic acid, *CAI* clinical activity index, *CRP* C-reactive protein, *CS* colonoscopy, *MES* Mayo endoscopic score, *PGE-MUM* prostaglandin E-major urinary metabolite; *s.d.* standard deviation.

### Relationship between variations in the MES, PGE-MUM, and CRP

**Figure 1:**
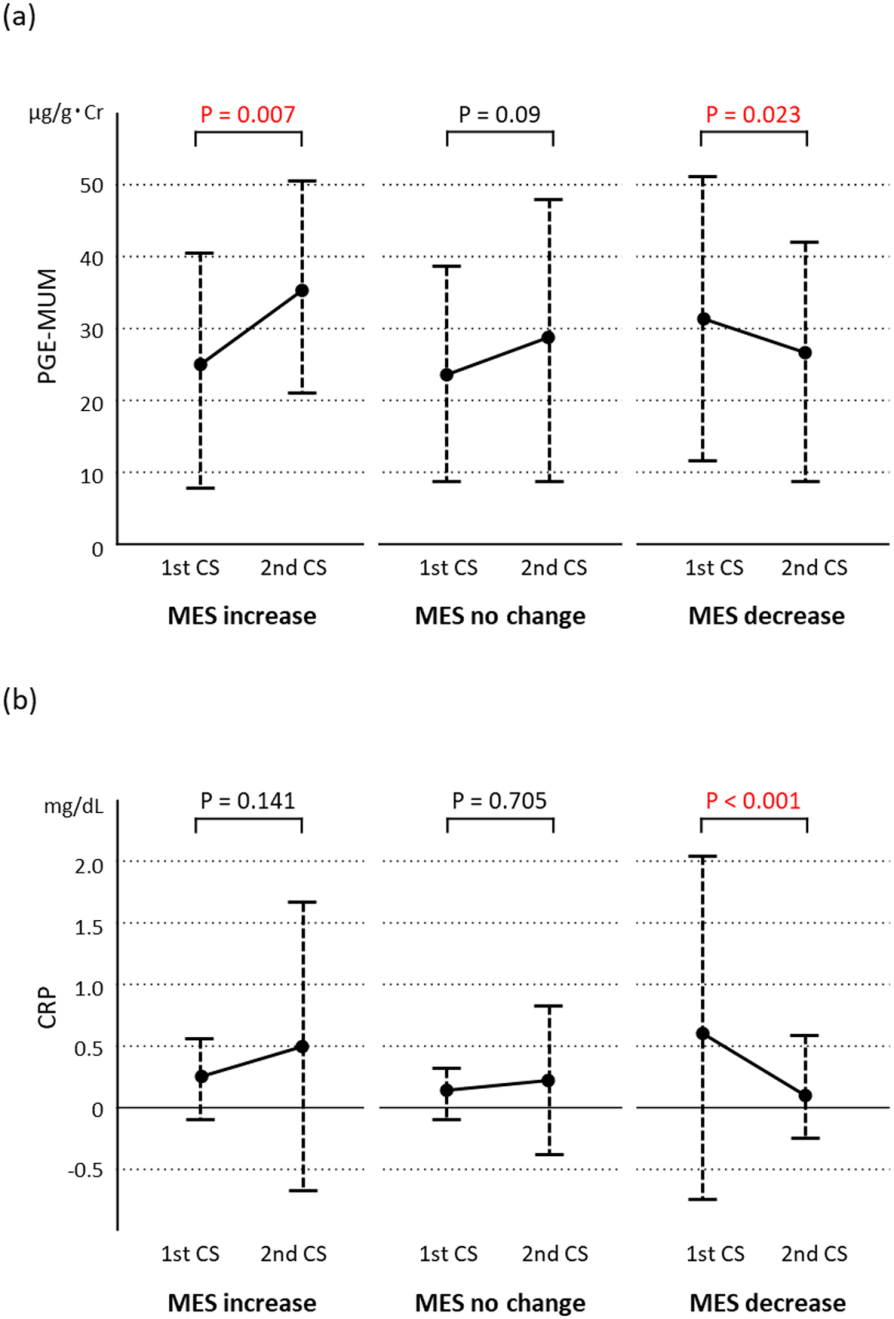
Varying levels of prostaglandin E-major urinary metabolite (PGE-MUM) (**a**) and C-reactive protein (CRP) (**b**) from the precedent colonoscopy (first CS) to the subsequent (second CS) in the Cohort 1 subgroups of Mayo endoscopic subscore (MES) increase, no change, and decrease.

In Cohort 1, variations in PGE-MUM and CRP values between the precedent and subsequent CS in the 3 MES subgroups were analysed (Fig. 1). The PGE-MUM values (Fig. 1a) showed a significant increase in the MES-increase subgroup and decrease in the MES-decrease subgroup (P = 0.007 and P = 0.023, respectively); no significant change was observed in the MES-no change subgroup (P = 0.090). The CRP values (Fig. 1b) showed a significant decrease in the MES-decrease subgroup (P < 0.001); however, no significant changes were noted in the MES-increase and MES-no change subgroups (P = 0.0141 and P = 0.705, respectively).

### Relationship between MH and variations in PGE-MUM and CRP

**Figure 2:**
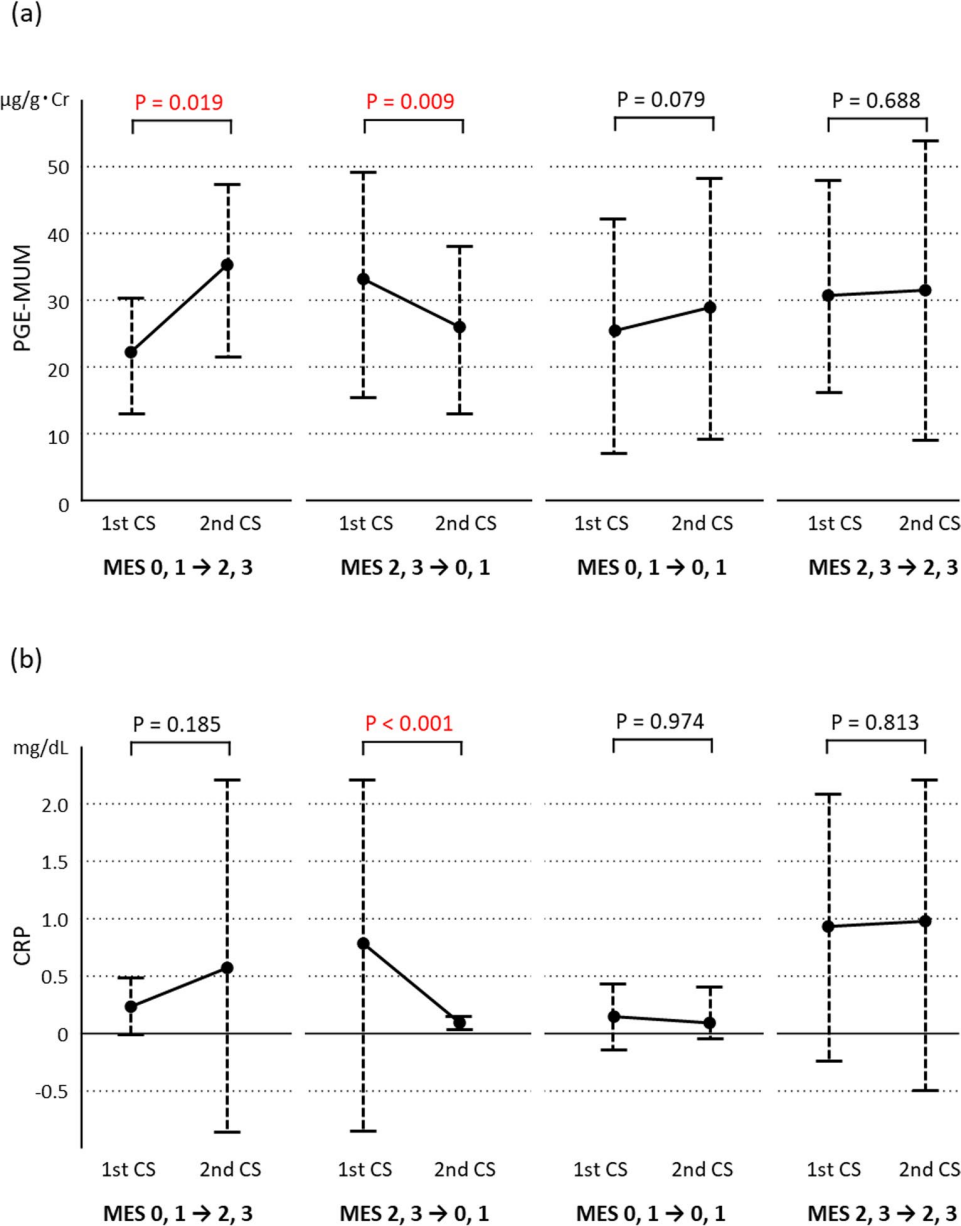
Varying levels of prostaglandin E-major urinary metabolite (PGE-MUM) (**a**) and C-reactive protein (CRP) (**b**) in the Cohort 2 subgroups, which reflected changes in MES scores, from the precedent colonoscopy (first CS) to the subsequent (second CS).

In Cohort 2, variations in PGE-MUM and CRP levels were analysed between the precedent and subsequent CS in the four subgroups (Fig. 2). In the subgroup that deteriorated from MES 0 or 1 to MES 2 or 3, the PGE-MUM value (Fig. 2a) increased significantly (P = 0.019), but the CRP value (Fig. 2b) showed no significant change (P = 0.185). In the subgroup that improved from MES 2 or 3 to MES 0 or 1, both the PGE-MUM and CRP values decreased significantly (P = 0.009 and P < 0.001, respectively). In the subgroups with no changes in MES 0 or 1 and MES 2 or 3, no significant differences were noted in PGE-MUM (P = 0.974 and P = 0.813, respectively) or CRP values (P = 0.974 and P = 0.813, respectively).

### Relationship between variations in S-MES, PGE-MUM, and CRP

**Figure 3:**
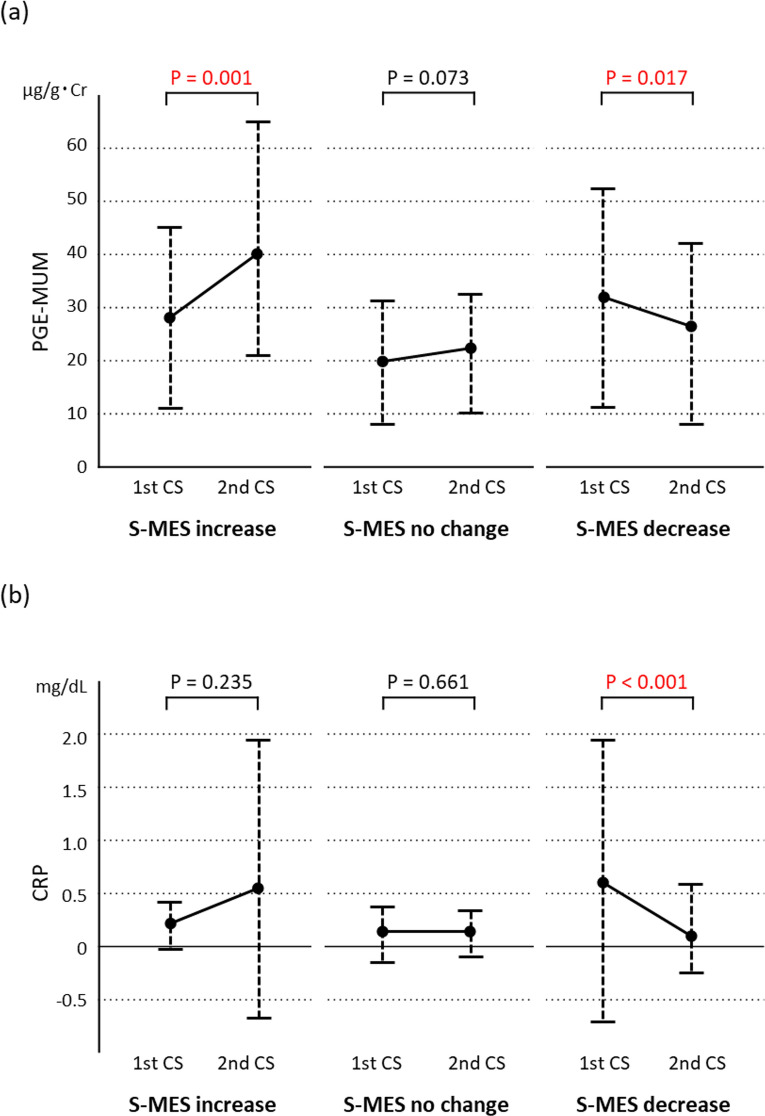
Varying levels of prostaglandin E-major urinary metabolite (PGE-MUM) (**a**) and C-reactive protein (CRP) (**b**) in the Cohort 3 subgroups of sum of Mayo endoscopic subscore (S-MES) increase, no change, and decrease from the precedent colonoscopy (first CS) to the subsequent (second CS).

In Cohort 3, variations in PGE-MUM and CRP were analysed between the precedent and subsequent CS in the three S-MES subgroups (Fig. 3). The PGE-MUM value (Fig. 3a) showed a significant increase and decrease in the subgroups of S-MES-increase and S-MES-decrease, respectively (P = 0.001 and P = 0.017); no significant change was observed in the S-MES-no change subgroup (P = 0.073). The CRP value (Fig. 3b) significantly decreased in the S-MES-decrease subgroup (P < 0.001); however, no significant changes were noted in the S-MES-increase (P = 0.235) or S-MES-no change (P = 0.661) subgroups (Fig. 3b).

**Figure 4:**
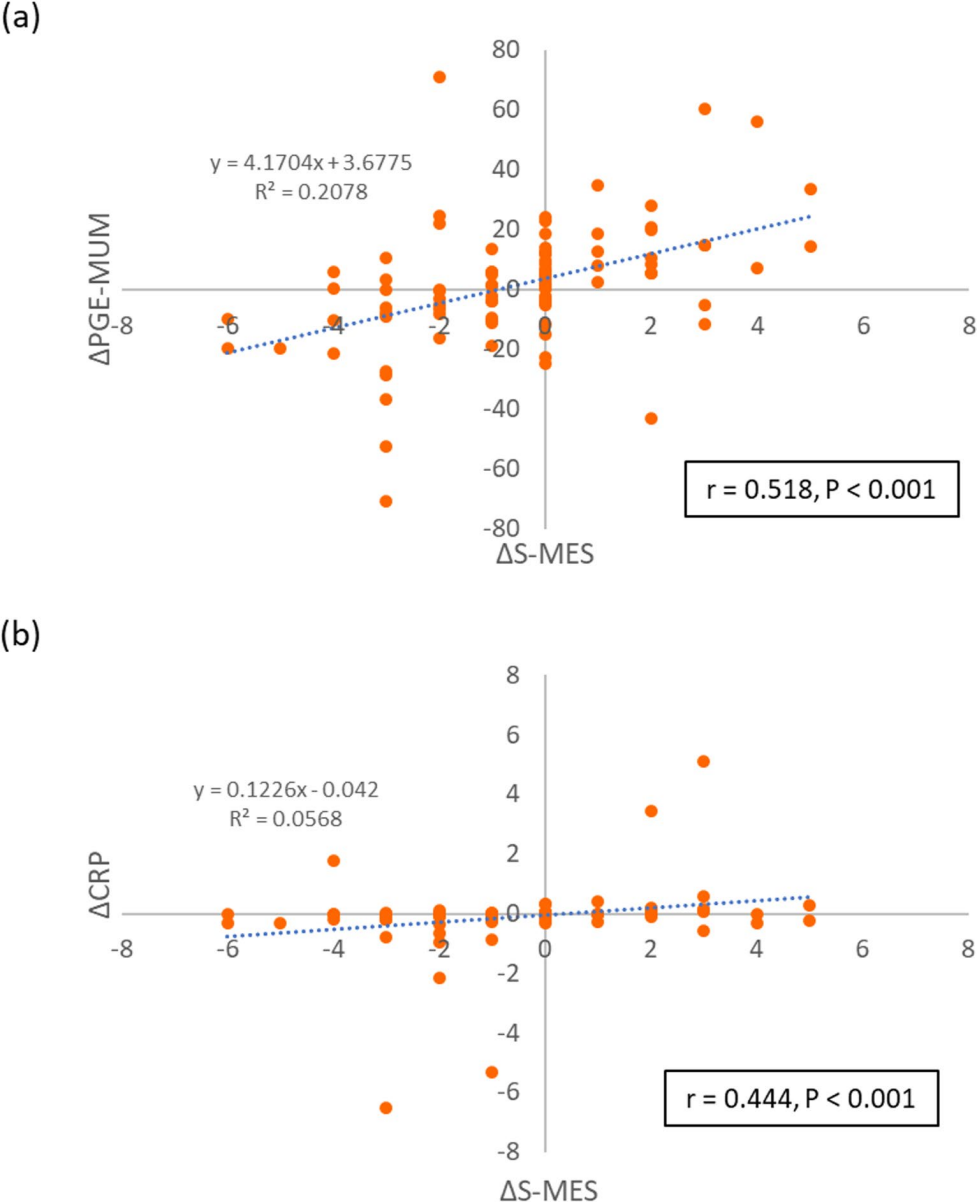
Correlations between variations in the sum of the Mayo endoscopic subscore (ΔS-MES) with variations in prostaglandin E-major urinary metabolite (ΔPGE-MUM) and C-reactive protein (ΔCRP). (**a**) Scatter plot of ΔS-MES and ΔPGE-MUM; ΔS-MES = (2nd–1st) S-MES; ΔPGE-MUM = (2nd–1st) PGE-MUM. (**b**) Scatter plot of ΔS-MES and ΔCRP; ΔCRP = (2nd–1st) CRP.

The correlations between a change in S-MES (ΔS-MES) with changes in PGE-MUM (ΔPGE-MUM) and CRP (ΔCRP) from the precedent to the subsequent CS were also analysed (Fig. 4). A significant correlation was observed between ΔS-MES and ΔPGE-MUM (r = 0.518, P < 0.001) (Fig. 4a), as well as between ΔS-MES and ΔCRP (r = 0.444, P < 0.001) (Fig. 4b). However, the correlation between ΔS-MES was stronger with ΔPGE-MUM than with ΔCRP.

## Discussion

When examining the utility of a biomarker for UC, it is critical to ensure the following: (1) reflection of endoscopic scores in cross-sectional evaluations; (2) prediction of clinical relapse; and (3) longitudinal variations in the endoscopic score. PGE-MUM has been proven to reflect endoscopic scores and predict clinical relapse in patients with UC^26,30,31^. This study aimed to examine whether longitudinal changes in PGE-MUM corresponded with changes in endoscopic scores of patients with UC.

PGE-MUM, a stable product of the metabolism of prostaglandin E2 (PGE2), is produced by the inflamed colon and is metabolized in the urine. The utility of PGE-MUM as a biomarker for UC was initially reported by Arai et al.^27^. PGE-MUM has been reported to reflect the endoscopic scores in paediatric patients with UC^28^. As PGE-MUM is present in urine, it possesses the advantages of hospital sample collection and submission, as well as unlikely detection failure by urinalysis. However, its levels may be elevated in cigarette smokers, patients with chronic lung disorders, and patients with cancer; hence, adequate care should be taken during analysis of such cases^32–37^.

In this study, PGE-MUM and CRP values showed significant changes that were parallel to the Cohort 1 of the MES-increase and MES-decrease subgroups. In Cohort 2, PGE-MUM values showed a significant increase and decrease in the Cohort 2 subgroups of remission phase to active phase and active phase to remission phase, respectively. In contrast, CRP values only showed a significant decrease with a decrease in endoscopic score (i.e., its value did not show a significant increase with an increase in endoscopic score). Therefore, we concluded that PGE-MUM reflects endoscopic scores with greater accuracy than CRP does, especially during exacerbations (increase in endoscopic score). Some studies reported the usefulness of CRP as a biomarker for UC; these studies defined MES 0 or 1 as MH, similar to the findings of our study^38–41^. In our previous cross-sectional study, MES 0, 1 was defined as MH, and receiver operating characteristic analysis of PGE-MUM to predict MH showed that the cut-off value was 30.9 μg/g·Cr and the area under the curve was 0.644 (95% CI 0.523–0.765), with a sensitivity of 48.5% and specificity of 78.0%^26^. However, most previously reported studies were cross-sectional studies, and only a few longitudinal observational investigations focused on endoscopic exacerbations of MH using MES. Although previous studies have used biomarkers for clinical trials or investigations with a focus on novel drugs, only a handful of analyses on biomarkers reflected endoscopic exacerbations. Endoscopic exacerbation of UC contributes significantly to its prognosis, and secondary treatment is recommended even in clinical remission. Therefore, determining MH by tracking the changes in PGE-MUM during UC treatment may be more beneficial than measuring CRP levels. Although data are not whown in Result section, we analyzed the correlation with the precedent CS data. The correlations of CAI with PGE-MUM and CRP were significant (r = 0.287, P = 0.004 and r = 0.258, P = 0.010, respectively). Although the correlations of MES with PGE-MUM and CRP were also significant, the correlation coefficient was greater for CRP than for PGE-MUM (r = 0.323, P 0.001 and r = 0.477, P < 0.001, respectively). However, since the biomarker values vary from UC patients to patients, we consider it important that the endoscopic findings and biomarker values fluctuate in parallel as shown in this study. The correlation plots diagram in Fig. 4 shows this parallel fluctuation.

Scoring indices, such as the MES and UC endoscopic index of severity employed in clinical practice only indicate the lesion displaying the most severe inflammation^42^. However, biomarkers may better reflect inflammation of the entire colon, which can be measured by S-MES. The S-MES possesses a broad range of scores, displaying variations in the inflammatory status with increased precision. A previous study reported that FC displayed a stronger correlation with S-MES (r = 0.86, P < 0.001) than with the conventional MES (r = 0.79, P < 0.001)^43^. PGE-MUM has also been proven to display a stronger correlation with S-MES than with MES (r = 0.413, P < 0.001 and r = 0.355, P < 0.001, respectively)^26^.

In this study, PGE-MUM values displayed significant parallel changes in Cohort 3 of the S-MES-increase and S-MES-decrease subgroups from the precedent to the subsequent CS. A significant correlation was also observed between changes in S-MES and changes in PGE-MUM values. In the conventional MES alone, the range was smaller at 0–3; however, in the S-MES, the range was as wide as 0–15. Thus, the S-MES may reflect a more detailed change in the endoscopic score than the conventional MES.

This study has several limitations. First, the investigation was conducted at a single centre with a limited number of enrolled patients. Second, PGE-MUM and CRP were the only biomarkers considered for comparison with the endoscopic scores of patients with UC. PGE-MUM was found to be superior to CRP in reflecting changes in endoscopic scores; however, FC, which has recently been employed in clinical practice and trials, was not incorporated into this study. In addition, biological data including FIT, leukocyte, platelet count, and serum albumin level was also insufficient. Third, as tissue healing has been regarded as a significant therapeutic goal for patients with UC in recent years, a comparative analysis between PGE-MUM and histological findings should have been done. Arai et al., examined the effectiveness of PGE-MUM as a biomarker by comparing it with histological scores in patients with UC^27^. Subsequent research should focus on comparing the utility of PGE-MUM as a biomarker with FC and histological findings in patients with UC.

In conclusion, variations in PGE-MUM values corresponded to changes in the endoscopic score of in patients with UC. In addition, the varying mucosal state of the colon was reflected more accurately by PGE-MUM levels than by CRP levels. Evaluation using PGE-MUM was considered to be a useful reflection of endoscopic exacerbations during the treatment of UC while aiming for MH.

## Methods

### Patients

This study’s the protocol was reviewed and approved by the Ethics Committee of Hamamatsu University School of Medicine (number 18-228) prior to research commencement. All procedures performed in studies involving human participants were in accordance with the ethical standards of the institutional research committee and with the 1964 Helsinki declaration and its later amendments or comparable ethical standards. All enrolled patients provided written informed consent to participate in the study.

A total of 60 patients with UC who underwent CS at the Hamamatsu University School of Medicine between April 2015 and October 2020 were enrolled in this study. Out of these, 40 patients were evaluated using three CS records (two intervals each) and the remaining 20 patients were evaluated using two CS records (one interval each); there were 100 intervals in total. The patients enrolled were diagnosed with UC based on a typical history, clinical features, and endoscopic and histological findings as per recent guidelines. Patients with IBD who were diagnosed with non-UC ailments, such as indeterminate colitis or unclassified IBD, as well as those with cigarette smoking habits, chronic fibrosing interstitial pneumonia, or malignant tumours were excluded, as these factors may have led to increased PGE-MUM levels^32–37^. The study participants were those for whom colonoscopy had been performed for routine surveillance or for clinical relapse of UC activity.

### Study design

**Figure 5:**
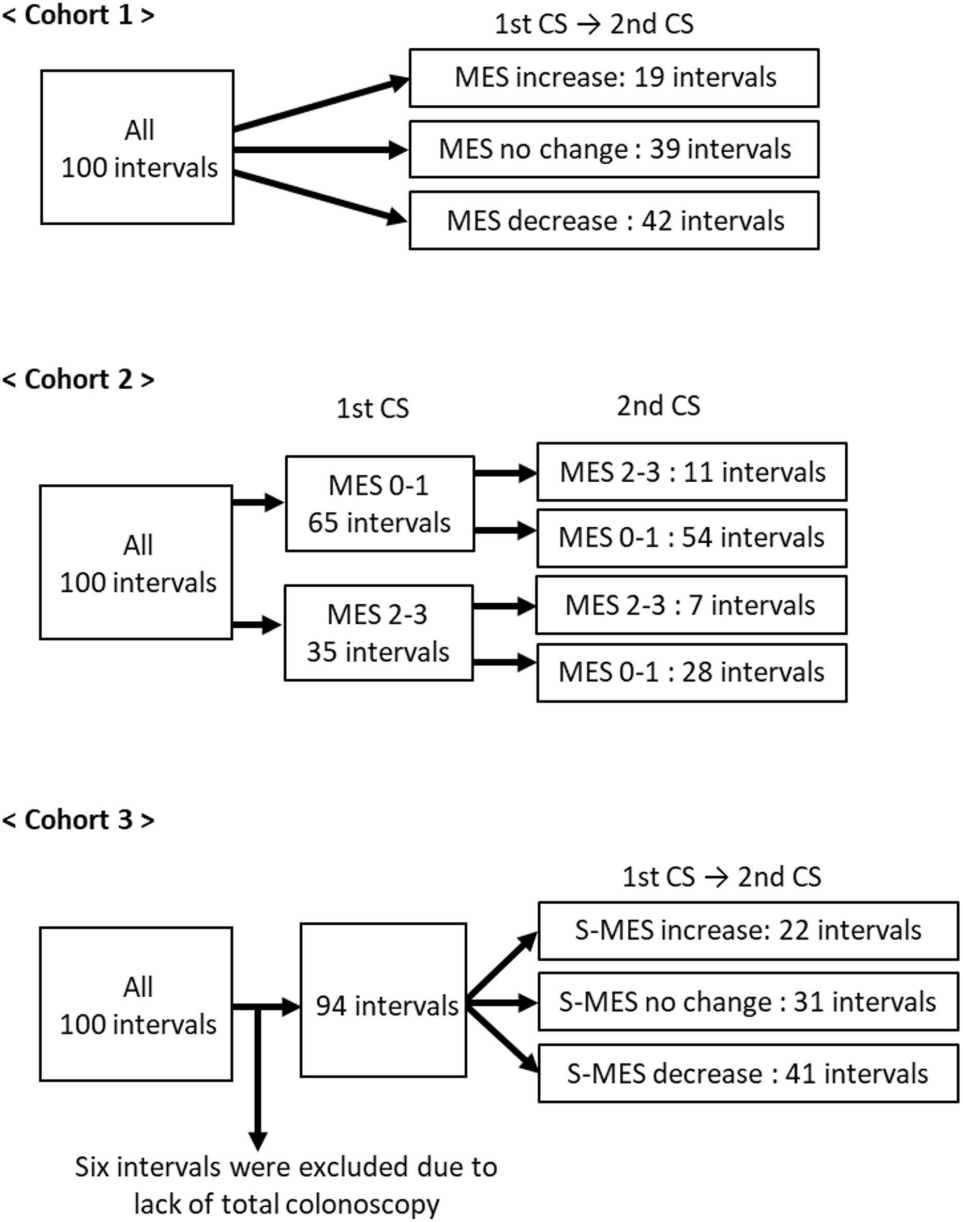
Study flow diagram. Three cohorts in the present study were divided as shown. In Cohort 1, 100 intervals were grouped as Mayo endoscopic subscore (MES) increase, no change, and decrease. In Cohort 2, groups of MES 0 or 1 and 2 or 3 were further subdivided as shown in the figure. In Cohort 3, 6 patients were excluded and the remaining 94 were grouped as sums of MES (S-MES) increase, no change, and decrease.

This prospective observational study aimed to longitudinally investigate whether PGE-MUM values changed with changes in endoscopic scores of patients with UC. The primary endpoint was whether the PGE-MUM value increased or decreased significantly as the endoscopic score increased or decreased during the two measurements over time. The secondary endpoint was whether the PGE-MUM value increased or decreased significantly with the exacerbation or achievement of MH. To evaluate these endpoints, we compared PGE-MUM and CRP values among three cohorts (Fig. 5).

In Cohort 1, changes in the Mayo endoscopic subscore (MES) at 100 intervals were used for the classification of the three subgroups: increase, no change, and decrease. Changes in PGE-MUM and CRP values were then evaluated for each subgroup. In Cohort 2, an MES of 0 or 1 was suggestive of endoscopic remission. Changes in PGE-MUM and CRP values were evaluated after categorizing MES values into four subgroups: MES 0 or 1 to 2 or 3 (remission phase to active phase); no change in MES 0 or 1 (stable remission phase); MES 2 or 3 to 0 or 1 (active phase to remission phase); and no change in MES 2 or 3 (stable active phase). In Cohort 3, the sum of MES (S-MES), an endoscopic score that reflects the mucosal state of the entire colon, was used for the classification of the three subgroups: S-MES increase, no change, and decrease. Changes in PGE-MUM and CRP values were then evaluated for each subgroup.

### Disease assessment

Disease intensity was evaluated using the clinical activity index (CAI) according to Rachmilewitz^44^. Clinical remission was defined as CAI ≤ 4, and a clinical response represented a decrease of ≥ 4 points relative to the baseline. Values of serum CRP were also used for disease assessment, and were measured on the same day as CS.

### Endoscopic assessment

CS was performed after bowel preparation was completed using a polyethylene glycol-based electrolyte solution, as previously reported in the literature^26,30,31^. The MES was employed to assess UC mucosal status and was defined as follows: 0, normal or inactive; 1, mild friability with erythema and decreased vascular pattern; 2, moderate disease with marked erythema, absence of vascular patterns, friability, and erosions; and 3, severe disease with spontaneous haemorrhage and ulceration^45^. MH was defined as MES of 0 or 1. Endoscopic scores were first evaluated by the physician who performed the colonoscopy, and then by five expert gastroenterologists (NI, S Tamura, TM, S Tani, KS). Any differences between these experts were resolved by consensus. To assess the entire mucosal state, S-MES was calculated by totalling the MES values of the five segments of the colon (ascending, transverse, descending, sigmoid, and rectum)^43^.

### PGE-MUM analysis

Spot urinary samples (≥ 0.8 mL) for the measurement of PGE-MUM were collected on the morning CS was performed. Samples were frozen at − 20 °C and delivered to the SRL Hachioji Laboratory (Tokyo, Japan), and the values were obtained with a ɤ-counter (Hitachi) using a bicyclic PGE-MUM radioimmunoassay (RIA) kit (Fujirebio, Tokyo, Japan). Biomarker levels corrected for urinary creatinine were used to avoid the effect of urine concentration.

### Statistical analysis

SPSS version 24.0 (IBM Armonk, New York, NY) and EZR (Saitama Medical Center, Jichi Medical University, Saitama, Japan) software were used for all statistical analyses of the data collected. The Wilcoxon signed-rank test was used to assess for significant changes in PGE-MUM and CRP values, and the Spearman’s rank correlation coefficient was used to assess the association between differences in the S-MES, PGE-MUM, and CRP changes in the precedent and the subsequent CS. A P-value of < 0.05 was considered significant.

## Data Availability

The data underlying this article will be shared by the corresponding author on reasonable request.

## References

[1] Podolsky DK (2002). Inflammatory bowel disease. N. Engl. J. Med..

[2] Annese V (2013). European evidence based consensus for endoscopy in inflammatory bowel disease. J. Crohns Colitis.

[3] Frøslie KF, Jahnsen J, Moum BA, Vatn MH (2007). Mucosal healing in inflammatory bowel disease: Results from a Norwegian population-based cohort. Gastroenterology.

[4] Ardizzone S (2011). Mucosal healing predicts late outcomes after the first course of corticosteroids for newly diagnosed ulcerative colitis. Clin. Gastroenterol. Hepatol..

[5] Colombel JF (2011). Early mucosal healing with infliximab is associated with improved long-term clinical outcomes in ulcerative colitis. Gastroenterology.

[6] Rutter MD (2004). Cancer surveillance in longstanding ulcerative colitis: Endoscopic appearances help predict cancer risk. Gut.

[7] Solem CA (2005). Correlation of C-reactive protein with clinical, endoscopic, histologic, and radiographic activity in inflammatory bowel disease. Inflamm. Bowel Dis..

[8] Zilberman L (2006). Correlated expression of high-sensitivity C-reactive protein in relation to disease activity in inflammatory bowel disease: Lack of differences between Crohn’s disease and ulcerative colitis. Digestion.

[9] Henriksen M (2008). C-reactive protein: A predictive factor and marker of inflammation in inflammatory bowel disease. Results from a prospective population-based study. Gut.

[10] Lok KH (2008). Correlation of serum biomarkers with clinical severity and mucosal inflammation in Chinese ulcerative colitis patients. J. Dig. Dis..

[11] Osada T (2008). Correlations among total colonoscopic findings, clinical symptoms, and laboratory markers in ulcerative colitis. J. Gastroenterol. Hepatol..

[12] Karoui S (2011). Correlation of C-reactive protein with clinical and endoscopic activity in patients with ulcerative colitis. Dig. Dis. Sci..

[13] Schoepfer AM (2009). Ulcerative colitis: Correlation of the Rachmilewitz endoscopic activity index with fecal calprotectin, clinical activity, C-reactive protein, and blood leukocytes. Inflamm. Bowel Dis..

[14] D'Haens G (2012). Fecal calprotectin is a surrogate marker for endoscopic lesions in inflammatory bowel disease. Inflamm. Bowel Dis..

[15] Schoepfer AM (2013). Fecal calprotectin more accurately reflects endoscopic activity of ulcerative colitis than the Lichtiger Index, C-reactive protein, platelets, hemoglobin, and blood leukocytes. Inflamm. Bowel Dis..

[16] Patel A, Panchal H, Dubinsky MC (2017). Fecal calprotectin levels predict histological healing in ulcerative colitis. Inflamm. Bowel Dis..

[17] Naganuma M (2020). Significance of conducting 2 types of fecal tests in patients with ulcerative colitis. Clin. Gastroenterol Hepatol..

[18] Nakarai A (2013). Evaluation of mucosal healing of ulcerative colitis by a quantitative fecal immunochemical test. Am. J. Gastroenterol..

[19] Mooiweer E, Fidder HH, Siersema PD, Laheij RJ, Oldenburg B (2014). Fecal hemoglobin and calprotectin are equally effective in identifying patients with inflammatory bowel disease with active endoscopic inflammation. Inflamm. Bowel Dis..

[20] Takashima S (2015). Evaluation of mucosal healing in ulcerative colitis by fecal calprotectin vs. fecal immunochemical test. Am. J. Gastroenterol..

[21] Ryu DG (2016). Assessment of disease activity by fecal immunochemical test in ulcerative colitis. World J. Gastroenterol..

[22] Shi HY (2017). Accuracy of faecal immunochemical test to predict endoscopic and histological healing in ulcerative colitis: A prospective study based on validated histological scores. J. Crohns Colitis.

[23] Hiraoka S (2018). Fecal immunochemical test and fecal calprotectin results show different profiles in disease monitoring for ulcerative colitis. Gut Liver.

[24] Shinzaki S (2017). Leucine-rich alpha-2 glycoprotein is a serum biomarker of mucosal healing in ulcerative colitis. J. Crohns Colitis.

[25] Nardone OM, Rispo A, Castiglione F (2020). Noninvasive monitoring of inflammatory bowel disease in the post COVID-19 era. Dig. Liver Dis..

[26] Ishida N (2020). Prostaglandin E-major urinary metabolite versus fecal immunochemical occult blood test as a biomarker for patient with ulcerative colitis. BMC Gastroenterol..

[27] Arai Y (2014). Prostaglandin E-major urinary metabolite as a reliable surrogate marker for mucosal inflammation in ulcerative colitis. Inflamm. Bowel Dis..

[28] Hagiwara SI (2017). Prostaglandin e-major urinary metabolite as a biomarker for pediatric ulcerative colitis activity. J. Pediatr. Gastroenterol. Nutr..

[29] Matsuno Y (2019). Measurement of prostaglandin metabolites is useful in diagnosis of small bowel ulcerations. World J. Gastroenterol..

[30] Ishida N (2020). Effect of ulcerative colitis duration on the usefulness of immunochemical fecal occult blood test result as a disease activity biomarker. Int. J. Colorectal. Dis..

[31] Ishida N (2020). Prostaglandin E-major urinary metabolite predicts relapse in patients with ulcerative colitis in clinical remission. Clin. Transl. Gastroenterol..

[32] Okayasu I (2014). Significant increase of prostaglandin E-major urinary metabolite in male smokers: A screening study of age and gender differences using a simple radioimmunoassay. J. Clin. Lab. Anal..

[33] Horikiri T (2017). Increased levels of prostaglandin E-major urinary metabolite (PGE-MUM) in chronic fibrosing interstitial pneumonia. Respir. Med..

[34] Johnson JC (2006). Urine PGE-M: A metabolite of prostaglandin E2 as a potential biomarker of advanced colorectal neoplasia. Clin. Gastroenterol. Hepatol..

[35] Kim S, Taylor JA, Milne GL, Sandler DP (2013). Association between urinary prostaglandin E2 metabolite and breast cancer risk: A prospective, case-cohort study of postmenopausal women. Cancer Prev. Res. (Phila).

[36] Zhao J (2015). Urinary prostaglandin E2 metabolite and pancreatic cancer risk: Case-control study in urban Shanghai. PLoS ONE.

[37] Kawamoto H (2019). Prostaglandin E-major urinary metabolite (PGE-MUM) as a tumor marker for lung adenocarcinoma. Cancers (Basel).

[38] de Bruyn M (2014). Neutrophil gelatinase B-associated lipocalin and matrix metalloproteinase-9 complex as a surrogate serum marker of mucosal healing in ulcerative colitis. Inflamm. Bowel Dis..

[39] Uchihara M (2017). Blood biomarkers reflect integration of severity and extent of endoscopic inflammation in ulcerative colitis. JGH Open.

[40] Yoon JY (2014). Correlations of C-reactive protein levels and erythrocyte sedimentation rates with endoscopic activity indices in patients with ulcerative colitis. Dig. Dis. Sci..

[41] Dranga M, Mihai C, Drug V, Dumitrescu G, Prelipcean CC (2016). A rapid test for assessing disease activity in ulcerative colitis. Turk. J. Gastroenterol..

[42] D'Haens G (2007). A review of activity indices and efficacy end points for clinical trials of medical therapy in adults with ulcerative colitis. Gastroenterology.

[43] Kawashima K (2016). Fecal calprotectin level correlated with both endoscopic severity and disease extent in ulcerative colitis. BMC Gastroenterol..

[44] Rachmilewitz D (1989). Coated mesalazine (5-aminosalicylic acid) versus sulphasalazine in the treatment of active ulcerative colitis: A randomised trial. BMJ.

[45] Schroeder KW, Tremaine WJ, Ilstrup DM (1987). Coated oral 5-aminosalicylic acid therapy for mildly to moderately active ulcerative colitis. A randomized study. N. Engl. J. Med..

